# Transferability of the EST-SSRs developed on Nules clementine (*Citrus clementina *Hort ex Tan) to other *Citrus *species and their effectiveness for genetic mapping

**DOI:** 10.1186/1471-2164-9-287

**Published:** 2008-06-16

**Authors:** François L Luro, Gilles Costantino, Javier Terol, Xavier Argout, Thierry Allario, Patrick Wincker, Manuel Talon, Patrick Ollitrault, Raphael Morillon

**Affiliations:** 1 INRA, Unité de Recherche GEQA, INRA San Giuliano, 20230 San Nicolao, France; 2 Centro de Genomica, Instituto Valenciano de Investigationes Agrarias, Valencia, Spain; 3 CIRAD AMIS, Montpellier, France; 4 UPR 'Amélioration génétique d'espèces à multiplication végétative', CIRAD, Montpellier, France; 5 Genoscope, CNS, Evry, France

## Abstract

**Background:**

During the last decade, numerous microsatellite markers were developed for genotyping and to identify closely related plant genotypes. In citrus, previously developed microsatellite markers were arisen from genomic libraries and more often located in non coding DNA sequences. To optimize the use of these EST-SSRs as genetic markers in genome mapping programs and citrus systematic analysis, we have investigated their polymorphism related to the type (di or trinucleotide) or their position in the coding sequences.

**Results:**

Among 11000 unigenes from a Clementine EST library, we have found at least one microsatellite sequence (repeated units size ranged from 2 to 6 nucleotides) in 1500 unigenes (13.6%). More than 95% of these SSRs were di or trinucleotides. If trinucleotide microsatellites were encountered trough all part of EST sequences, dinucleotide microsatellites were preferentially (50%) concentrated in the 5' 100th nucleotides. We assessed the polymorphism of 41 EST-SSR, by PCR amplification droved with flanking primers among ten *Citrus *species plus 3 from other genera. More than 90% of EST-SSR markers were polymorphic. Furthermore, dinucleotide microsatellite markers were more polymorphic than trinucleotide ones, probably related to their distribution that was more often located in the 5' UnTranslated Region (UTR). We obtained a good agreement of diversity relationships between the citrus species and relatives assessed with EST-SSR markers with the established taxonomy and phylogeny. To end, the heterozygosity of each genotype and all dual combinations were studied to evaluate the percentage of mappable markers. Higher values (> 45%) were observed for putative Citrus inter-specific hybrids (lime lemon, or sour orange) than for Citrus basic true species (mandarin, pummelo and citron) (<30%). Most favorable combinations for genome mapping were observed in those involving interspecific hybrid genotypes. Those gave higher levels of mappable markers (>70%) with a significant proportion suitable for synteny analysis.

**Conclusion:**

Fourty one new EST-SSR markers were produced and were available for citrus genetic studies. Whatever the position of the SSR in the ESTs the EST-SSR markers we developed are powerful to investigate genetic diversity and genome mapping in citrus.

## Background

Simple Sequence Repeats are tandem repeat sequences that are quite abundant in eukaryotes genomes [1]. Numerous genomic libraries enriched in SSR have been established from many plant species [2-5]. Those repeat sequences also called microsatellites (MS) present a higher level of polymorphism and higher expected heterozygosity when compared with to other dominant (AFLP and RAPD) or codominant markers (RFLP) [6]. Since SSRs are ubiquitously present in genomes with randomly occurrence, they are communally used as genetic markers in many different plant species to unravel the interspecific and intraspecific diversity [7-10].

In citrus, the number of published markers of genomic SSRs is still limited [11,12]. Those markers were used for genetic diversity assessment and for germplasm management [13,14]. A high-density microsatellite consensus map is still lacking. The major goal of genetic mapping is to localize genes or QTLs, involved in traits of interest that are linked to molecular markers. Those molecular markers can be used as a starting point for gene identification or to reduce schemes of selection. One other way to address this aim is to develop markers directly localized in the coding sequences. ESTs (Expressed Sequence Tags) derived from cDNA libraries obtained from the genome expression have been investigated for microsatellite screening, in barley [15], wheat [16], rice [17], citrus [18,19], sugarcane [20] and grape [21]. It is assumed that those SSRs markers should enable to assess the molecular evolution of the genes in which they are positioned. Indeed, it has been observed that in ESTs, the flanking region of SSRs are more conserved and can also be found in related genera [22]. Thousands of EST-SSRs were identified in numerous species such as grape and cereal. A high level of transferability was noted between rice, wheat and barley [17]. In citrus, thousands of ESTs are now available in databases. Recently, using public sequence databases resources, Chen et al. [23], published the characterization of 56 EST-SSR markers identified among 2295 citrus ESTs, mappable in a progeny obtained from a cross between sweet orange (*Citrus sinensis *L. Osb.) and trifoliate orange (*Poncirus trifoliata *L. Raf.). If those two genotypes represent important resources of agronomical characters for rootstock and cultivar improvement scheme, numerous other citrus species offer a large panel of specific traits interesting breeders or consumers. For example, Clementine (*Citrus clementina *Hort. Ex Tan.) is a model citrus crop in Mediterranean area and sour orange (*C. aurantium *L.) or Cleopatra mandarin (*C. reshni *Hort. Ex Tan.) are tolerant to abiotic constraints such as salt stress or calcareous soils [24]. Citrus as many fruit trees have a juvenility period with around 5 years of duration limiting the possibility to study the allelic segregation on a second generation of hybrids (F2 or BC). Consequently citrus genetic maps are established on F1 progenies at interspecific [25], and intergeneric levels [26-31]. To maximize the potential for the development of EST-SSR based maps we need to investigate the polymorphism and the heterozygosity of these markers in different combined genotypes at the origin of F1 progenies. Another point of reflexion concerning the polymorphism of SSRs in EST is the usefulness of the derived markers such as STMS (Sequence Tagged MicroSatellite) in cultivar distinctness and in relationships between varieties and species. The particular position of these SSRs inside coding sequences may question the genetic diversity information that we can extract from those markers related to the putative influence of the selection on the SSR polymorphism.

In a full-length clementine (*Citrus clementina*) ESTs database [19], we looked for SSR markers. Screening of 37 000 ESTs allowed us to identify about 1600 SSRs. We report here the outline investigation of the polymorphism of EST-SSR among a set of 16 citrus species covering a wide range of citrus genetic diversity. We assessed also the mappability of these markers on our different progenies established for heredity studies. The effect of repeated motif length (dinucleotide or trinucleotide) and their position on the cDNA sequence, on their polymorphism are also discussed.

## Methods

### SSR detection

SSR detection was undertaken on 11632 non-redundant sequences generated by the StackPACK application homepage [32] from 37 000 ESTs obtained from Nules clementine. The MIcroSAtellite identification tool (MISA) [33] was used to perform the search of 2 to 6 nucleotide motif repeats into the unigene dataset. Dinucleotide SSRs were identified with a minimum of six repeats and the other types of SSR with a minimum of five repeats. The maximum interruption between 2 SSRs to consider a SSR as a compound one was set at 100 nucleotides. Perl script modules linked to the primer modelling software Primer3 [34], were used to design primers flanking each SSR region found. The primer product size range was chosen between 100 and 280 nucleotides. The optimum size of primers was set to 17 nucleotides (range from 15 to 23 nucleotides) with an optimum melting temperature of 56.0°C (range from 50 to 63°C). When possible, 3 pairs of primers were picked for each STMS. The localization of SSRs in comparison with the coding sequence was estimated by BLASTx analysis realised during initiation of the Clementine EST Database (ESTtik, CIRAD, Montpellier, France) for assessing putative function to the unigene sequence. The codon sequences were translated in nucleotide sequences and then the SSR position related to the CDS was elucidate and detailed as following: in 5'UTR, in CDS or in 3'UTR.

### Plant material

Sixteen citrus genotypes were investigated for microsatellite screening. Thirteen varieties from 10 species were chosen to represent the *Citrus *genus (Table 1). One accession of the two other true citrus genera, *Fortunella marumi *and *Poncirus trifoliate *and a related wild genus, *Severinia buxifolia*, completed the citrus sample set. All those accessions are maintained in the INRA CIRAD citrus depository at San Giuliano (Corsica, France).

**Table 1:** Citrus accessions used in this study for STMS screening maintained at the Corsican citrus germplasm.

Latin name	Commun name	Varietal name	Accession number
*Citrus clementina *Hort. ex Tan.	clementine	Nules	SRA 498
*Citrus sinensis *(L.) Osb.	sweet orange	Washington navel	SRA 555
*Citrus reshni *Hort. ex Tan.	mandarin	Cleopatra	ICVN 0110066
*Citrus deliciosa *Ten.	mandarin	Willow leaf	SRA 133
*Citrus aurantium *L.	sour orange	Morocco	ICVN 0110038
*Citrus paradisi *Macf.	grapefruit	Marsh	SRA 293
*Citrus medica *L.	citron	Corsican	SRA 613
*Citrus aurantifolia *(Christm.) Swing.	lime	Mexican	SRA 140
*Citrus limettioïdes *Tan.	lime	Brazil sweet	SRA 697
*Citrus limon *(L.) Burm.	lemon	Lisbon Foothill	SRA 196
*Citrus maxima *(Burm.) Merr.	pummelo	Sans pépin	SRA 710
*Citrus maxima *(Burm.) Merr.	pummelo	Pink	SRA 322
*Citrus hystrix *D.C.	combava	Kindia	SRA 630
*Poncirus trifoliata *(L.) Raf.	trifoliate orange	Rubidoux	ICVN 0110128
*Fortunella japonica *(Thunb.) Swing.	kumquat	Marumi	SRA 482
*Severinia buxifolia *(Poir.) Ten.	box orange		ICVN 0110249

### EST functional annotation

Functional annotation of ESTs was based on Gene Ontology (GO) annotation [35], and performed of with BLAST2GO [36]. B2G parameters were: NCBI non-redundant DB for BLAST search, 20 hits maximum for BLAST result, 100 nt as minimum HSP-length to retain putative annotating hits and default Evidence Code Weights for Gene Ontology annotation that assigns high ECWs to experimental-based and curate annotations while penalized electronic and non-curate annotations. Minimum values for BLAST e-value and % similarity of the BLAST result were e-06 and 55% respectively and ultimate annotation cut-off value was set to 55.

To provide a broad representation of the distribution of gene product functions, the ESTs were organized in sets according to broad GO ontology categories, as described by the GO Slim Classification for Plants developed at TAIR. GOSlim annotations of the *Citrus *ESTs were also generated with the B2G software, using the plant GOSlim mapping tool provided in TAIR. The GO Slim classification was performed for both the whole collection of 37 000 ESTs and the subset of sequences carrying SSRs.

### SSR polymorphism analysis

Total DNA was extracted from leaf samples according to the method developed by Doyle and Doyle [37]. Amplifications were performed according to Kijas et al. [11] in a thermocycler (PTC 200, MJ Research) using 10 ng of DNA, 0.5 μM of each primer and 0.8 unit of *Taq *polymerase (Goldstar, Eurogentec). The annealing temperature was fixed for all primer pairs at 55°C (this condition was taking account during the primer designing). Separation of alleles was performed on a 6% polyacrylamide sequencing gel containing 7 M urea in 0.5× TBE buffer at 60 W for 2 h to 3 h. Three microliters of PCR product was mixed to an equal volume of loading buffer containing 95% formamide, 0.25% bromophenol blue and 0.25% xylen cyanol, and 10 mM of EDTA. This mixture was heated for 5 min at 94°C to denature the DNA before loading. Gels were stained with silver nitrate following the protocol detailed by Chalhoub et al. [38], for gel electrophoresis analysis and by comparison with the 10 bp DNA standard ladder (Invitrogen).

### Genetic diversity and data analysis

Four parameters of diversity were estimated for each locus corresponding to a subset of 39 SSR markers: percentage of polymorphic loci, the mean number of alleles per locus, observed heterozygosity (H_0_), and the identification rate (IR). H_0 _was estimated for each type of EST-SSR marker. IR represents the degree of polymorphism of each marker suitable for genotype distinctness and was calculated as $\frac{1}{n}\sum_{i=1}^{n}Pi$ where *Pi *is the rate of identified genotypes across all individuals at *i *locus and *n *is the number of observed loci. The value of IR varies between 1 (all the individuals are distinct at all loci) and 0 (all individuals have a same molecular profile at any locus). An ANOVA was applied as statistical analysis to test the effect of the SSR features on diversity parameters.

To determine the genetic diversity structure and relationships between species we scored the SSR profile at 41 loci for each citrus sample by coding the presence (1) and the absence (0) of each allele. Genetic distance between each citrus genotype was estimated by calculating the Dice dissimilarity index [39]. A dendrogram was constructed with the Neighbour joining method [40]. This analysis was performed with the "DARwin" software developed by CIRAD (Montpellier, France). We have calculated the percentage of heterozygous loci of each of the 15 genotypes (*Severinia buxifolia *was not included in this analysis) and also the percentage of polymorphic and monomorphic heterozygous loci between each pair of genotypes. The percentage of mappable loci in each hypothetical genotype association was estimated by the addition of the rate of heterozygous loci from two parents and avoiding to taking account twice the commune markers.

## Results

### EST-SSRs frequency and GO representation

**Figure 1:**
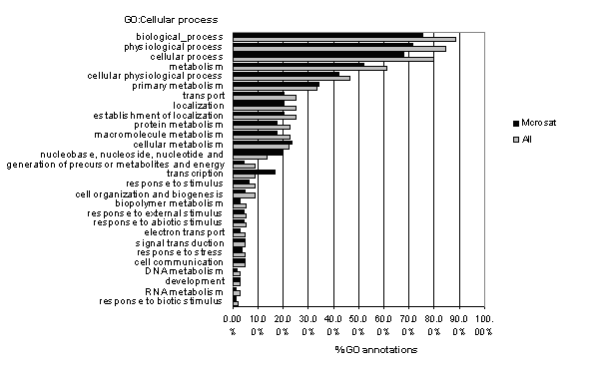
Comparison of the unigenes distribution in MIPs function categories between the citrus EST collection and the ESTs that contain SSR.

1692 SSRs (excepted mononucleotide unit) were identified among 11 391 unigenes from 37 000 EST clones. We first analyzed the type nucleotide repetition in the SSRs. Some unigenes contained more than one microsatellite sequences and at the end, 1501 unigenes (13%) had at least one SSR. Functional characterization of ESTs was performed assigning Gene Ontology annotations [35], with the BLAST2GO software [36]. To provide a general representation of the annotation, the Slim GO Classification was obtained (see Materials and Methods), for both the whole set of ESTs and the subset displaying SSRs. ESTs with SSRs were present in every major Slim GO category, and no significant differences could be found with respect the whole EST collection (Fig. 1).

The different SSRs found among our Clementine EST library and their frequency were: the most common trinucleotide repeats (53.9%) followed by dinucleotide repeats (37.6%), tetranucleotide repeats (3.7%), hexanucleotide repeats (2.4%) compound repeats (2%) and the less abundant pentanucleotide repeats (0.4%).

### Distribution of di or trinucleotide SSRs on ESTs

**Figure 2:**
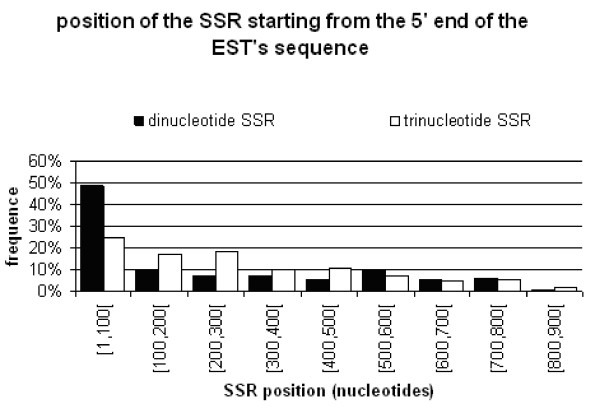
Position of the dinucleotide and trinucleotide SSRs from 5' end EST sequences.

The SSRs display preferential location along EST sequences from clementine EST database [19] was, with a high concentration of these before the 100^th ^nucleotide from the 5' extremity (75%). The analysis of the SSR type showed a difference on distribution along the EST sequence (Fig. 2). Dinucleotide microsatellites were located preferentially at the beginning (5'part) of the cDNA (50% of the total were located before the 100^th ^nucleotide) and in the UTR (75%). Trinucleotide SSRs were less concentrated at the beginning of the 5' terminal region of the cDNA sequence (25%) when compared to dinucleotide SSRs. Microsatellites were localized either inside, either outside the translated region (TR). Since the absence of a stop codon in some cDNA sequences (the sequencing was not complete in the 3' extremity), it wasn't possible to detect any translated sequences or ORFs (open reading frame) for the cDNA sequences corresponding to the EST-SSR markers N° 16, 21, 26, 34 and 43. For EST sequences where the TR was detected, dinucleotide SSRs were preferentially concentrated (75% of them) in untranslated regions (UTR). Trinucleotide microsatellites were equitably distributed inside and outside the TR of the ESTs (48% and 52% respectively).

### Development of EST-SSR markers

A set of 48 pairs of primers was randomly chosen among the 1692 microsatellites that matched with identified genes sequences from nucleic acid data bases (EMBL or NCBI) to amplify 23 dinucleotide SSRs and 25 trinucleotide SSRs. Among them, 7 did not amplify even clementine suggesting that the selected primers were not adapted or that the PCR product was too large to be amplified. 41 primer pairs amplifying DNA fragment in Clementine were presented in Table 2. In order to check the redundancy or the novelty of those markers, we compared by BLASTn the sequences of EST clones supporting the development of EST-SSR markers in clementine with those published by Chen et al [23]. We have not detected any similarity between both sets of markers. The amplified DNA profile of EST-SSR markers N°115 and N°482 were multi-bands suggesting genome duplications of corresponding genes or nonspecific PCR amplification. We have also compared the expected size of DNA fragment containing each SSR and flanked by primers (calculated from the EST sequence), and the size of corresponding amplified fragments from genomic DNA of Clementine (estimated on gel electrophoresis) (Table 2). The observed PCR product sizes were mainly equal to the expected ones with differences minus than 10 nucleotides. These small variations could be associated to errors during ESTs sequencing or in size estimation on gel electrophoresis. For 6 EST-SSR markers (N° 21, 25, 34, 203, 228 and 430) variations were greater than 30 nucleotides with a maximum of 270 bases of increase of the DNA fragment size for marker N°430. In those markers, we suspected the presence of introns in the amplified DNA fragments.

**Table 2:** Primers sequences of a random selection of 41 EST-STMS.

SSR name	EST Accession number	SSR Type	Forward Primer Sequence (5' to 3')	Reverse Primer Sequence (5' to 3')	Expected Product size (b)	Observed product size (b)	SSR localization
5	DY262823	(AG)11	AAGGCATAGCAAAGAAGCCA	CTTGGGCCATCATCTACTGG	203	204/236	cds
10	DY263095	(TC)9	TCAAAGTTGATTTTCATTTGCC	GGGAACATCATAGTCGGTGC	165	178/180	5' UTR
16	DY264179	(TC)13	ACCTGAGCCCTTTTTGGTTT	GCCAGATCAAGGCTCAAATC	136	133/136	nd.
20	DY264355	(AT)7	AAAAACACCTGTGGGACAGC	TAAACACTCCAGGCACCCTC	123	125	5' UTR
21	DY264533	(TC)8	TGATCAGCAACCAATAACCG	AGTCCGTCGTTTGTGATGTG	240	240	nd.
25	DY264633	(TC)6	CGGTCAGGTCCTCACATACA	TGATCTTCTTCGCCTCCATT	206	350	5' UTR
26	DY265129	(TC)6	GTTCTCCCCTTCCCTCTCTG	CCAATGATGAAAGCCAAACA	268	300	nd.
34	DY265633	(TA)6	TTATGCTCCGGCTGCTTAGT	AAAAGCCACTCGTTACACGG	165	166	nd.
43	DY266190	(TA)6	CGAACCACTCCCCATCTCT	TGATGGTGGTGTTCTCCTTG	130	330	nd.
115	DY274953	(TA)6	CCCCCTCTTCTTTCACACAA	GGTGAGCAGCCATCTTCTTC	136	136/140	5' UTR
159	DY280390	(GA)10	TTTTTGGCTTTCTGGGTTTG	GCTCCACTGGGATAGCTGAG	243	multibands	5' UTR
282	DY294129	(CT)6	GGACCAGAAGCAGGTTTTGT	AAAGAGCGATGACCCAAAAA	201	187/201	cds
295	DY294759	(TC)6	CACCTTCTCAGGCAATCTCC	TTGAGCGATGTGAAGAGGTG	133	134	cds
482	DY296883	(GA)10	CCCCCTCTTTTTCTCTTCCA	TTCTGGGCTGGTAGGTTCAG	215	210/214	cds
652	DY262841	(GA)11	TCTTCTGCTGGAAACAAGCC	TGGAAGAGAAGAAACGGTGG	221	multibands	5' UTR
817	DY287851	(TA)17	CCCAGCTTCCAGAGAAGAGA	GTCAAGAATCAAGCAGGCGT	195	195/219	5' UTR
830	DY284947	(TC)6	TTCATGGCAGCTTGAGTTTC	TTGGTTTCTTTTGGGGATCA	197	197/199	cds
1527	DY292105	(TC)6	GCGCGATCACTCTCTTTCTT	ATCGGGTTTGGATTAGGGAC	114	114/116	cds
32	DY265504	(CAG)6	CAGATCCTATTGCAGAGGCA	GCCCATTTGTATTGCCATTT	175	178	5' UTR
67	DY268562	(AGC)5	ATGTGGCTCCCTCTTCTCCT	GTGCATAACTGGGCCGTACT	175	192/195	5' UTR
92	DY272212	(ATC)5	CGCAGCTTTTGCATGTTTTA	TGCTGCTAACCCACAGACAG	242	253	cds
93	DY272212	(CTT)5	TGCATTTTCACCTCAGCAAC	GGGAGAGAGAGAAAGCCAGC	212	210	cds
116	DY274953	(AGA)7	GAATTGGGAGGACGAACTGA	CGAGCCCTAGACAGAGATGG	252	249/252	cds
117	DY275245	(TCA)6	AACAAACCCAGAACACTGCC	TGAGTGTGGGCGTAGATTGA	108	108	5' UTR
121	DY275927	(TAA)9	TCCCTATCATCGGCAACTTC	CAATAATGTTAGGCTGGATGGA	181	180	3'UTR
137	DY277386	(CAA)5	CGTCTTGCTCGCTGTATCTG	TCGCTTTTGGGATTTGAGAC	166	166	5' UTR
154	DY279967	(GCC)5	AAGCCTCAAGTCAAGGCAAA	GCCCCATTTTGTATGGAGTG	107	105/108	5' UTR
164	DY281040	(GCC)5	GTTTTCAGCTGGATTCGAGG	CACGTGTCCTCCTGGAACTT	180	181/187	cds
175	DY281748	(CAG)6N38(GCA)6	ACAGCAACCCCAGTCACTCT	CGCTCCTCGATTTGAAGAAG	252	252	5' UTR
179	DY282259	(CTT)5	TTCTCTCTCTCGAGCTTCGC	CCCAATCATCCTCCGTTAGA	210	220	cds
196	DY283426	(CGC)5	TCTTCTTCCCTGCTTTTCCA	ATCAAGGAGATCCATGTGGG	274	275	cds
203	DY284275	(CTT)5	CTTCACAACCAAGGCCATTT	CTGTGTGCGAGCGTATCACT	205	205	cds
228	DY286984	(GAG)5	TGAAGGTGCTAGGATTGGCT	CGGACACTCAAAAGCTGACA	238	508	5' UTR
238	DY288340	(TTC)5	CATGTTTCATTGCAAATGCC	TCTGGACATTCCATCACCAA	272	370	5' UTR
338	DY299973	(CTT)11	TTTCTAAAATTTCCTTCATGGC	CAGGTGAAATCTCATCGCCT	204	204/216	5' UTR
418	DY274485	(AAT)5	AAAACAAACGCCACCTAAATG	CAGCAGCTGAAAACACCTGA	134	135	3'UTR
430	DY275609	(AAT)7N15(AGC)7	CCGATACAGCACAAAGCAAA	TGGAAAGAGAGAAGCCAAGC	129	130	cds
432	DY275609	(GAG)5	GAGCTCAAAACAATAGCCGC	CATACCTCCCCGTCCATCTA	226	330	cds
818	DY287851	(TCT)6	GTAGATTCGTTCAAGGCCCA	GTGAAGCTGGAAGAGATGGC	134	135	5' UTR
1210	DY295001	(ATC)5	GCCAAAATGCATGTTCAAGA	GTGCCAATGATGATCACGTC	175	183	5' UTR
1388	DY289396	(GGA)6	AAAACAAAGCACCCAGATCG	ACGGCAGCAACGAGATAAGT	138	139	cds

Both the expected PCR product size and observed product size are base number (b), multibands mean a non specific amplification or multilocus profile. Cds: Coding sequence; 3'UTR or 5'UTR 3' end or 5' end of untranslated region

The 39 single EST-SSR markers were used to amplify the DNA of 16 genotypes representing a wide genetic variation of the Corsican citrus germplasm. Amplifications were successful for all the citrus genotypes with all primer pairs excepted for 3 markers which did not amplify any DNA fragment for Box orange (*Severinia buxifolia*). This genotype was considered to a member of the *Citrinae *subtribes as true citrus genera (*Poncirus*, *Fortunella *and *Citrus*).

**Figure 3:**
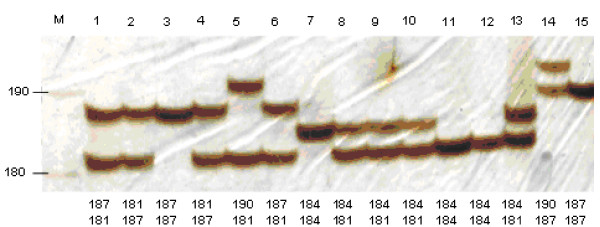
**Polymorphism of the trinucleotide microsatellite (EST164 STMS) among citrus genotypes detected by silver nitrate staining gel electrophoresis.** Below the photography size allelic interpretation of each genotype is detailed. The order of the sample is the following: Invitrogen 10 bp ladder (Lane M), 'Nules' clementine (lane 1), 'Washington Navel' sweet orange (lane 2), 'Cleopatra' mandarin (lane 3), 'Willow leaf' mandarin (lane 4), 'Morocco' sour orange (lane 5), 'Marsh' grapefruit (lane 6), 'Corsican' citron (lane 7), 'Mexican' lime (lane 8), 'Brazil sweet' lime (lane 9), 'Lisbon Foothill' lemon (lane 10), 'Sans pepin' pummelo (lane 11), 'Pink' pummelo (lane 12), 'Kindia' combava (lane 13), 'Rubidoux' trifoliate orange (lane 14), 'Marumi' kumquat (Lane 15).

Observed size variations in amplified DNA fragments were always correlated to the size of the repeated sequence unit of each SSR suggesting that the polymorphism was only related to the difference of repetition number of SSR. On the figure 3 is represented the polymorphism detected with the trinucleic microsatellite marker N°164. Note that the size differences between each DNA fragments were equal or multiple of 3 (181/187 bases, 181/190 bases, 184/181 bases for respectively Nules clementine, Morocco sour orange and Mexican lime). The sequencing of these different SSR alleles confirmed that the size variation was due to the difference in the number of repeats unit.

### Position and SSR type effect on polymorphism

Polymorphism, number of allele per locus and number of genotypes per locus for the 16 dinucleic and 23 trinucleic SSRs when compared to their respective position on the EST sequence (Table 3). Without any distinction about the type of SSR we have observed an effect of the SSR position on the polymorphism. The polymorphism obtained was greater when SSRs were in UTRs (86% versus 67%) whatever the type of repeats. Considering only the type of repeated unit, differences were also observed. If the percentage of polymorphic loci was quite similar between trinucleic SSRs (83%) and dinucleic SSRs (80%), for the two last indicators of marker diversity the effect of unit repeat was significantly different. Dinucleic repeated units revealed significant higher polymorphism than trinucleic repeated units with 7.3 versus 4.1 alleles per locus (P = 0.015), and 0.61 versus 0.29 for the IR (P = 0.010). If we combine parameters, type and position, differences were particularly important for SSRs localized in UTR. In this situation, dinucleic repeats had a number of alleles per locus greater than trinucleic repeats (7.9 versus 4.4) and a rate of identification 2 fold greater (0.66 versus 0.29). The higher value of alleles per locus for dinucleic SSRs could be related to the higher percentage of heterozygous loci (54% versus 29% for trinucleic SSRs).

**Table 3:** Comparison of polymorphism parameters between dinucleotide and trinucleotide EST-SSR located in untranslated region (UTR) or in translated region (TR)

	SSR type	SSR in TR	SSR in UTR	Total
% of polymorphic loci	dinucleotide	67	83	80
	trinucleotide	67	93	83
	indistincte	67	86	82
Number of allele per locus	dinucleotide	5,3	7,9	7,3
	trinucleotide	3,6	4,4	4,1
	indistincte	4	5,9	5,25
Identification rate per locus	dinucleotide	0,44	0,66	0,61
	trinucleotide	0,31	0,29	0,29
	indistincte	0,34	0,44	0,41

### EST-SSR markers for genetic mapping

Heterozygosity of genotypes is a key component for genetic mapping on F1 progenies classically used for citrus genetic mapping. Based on a unique F1 progeny obtained from a cross between two heterozygous genotypes it is possible to develop a genetic map for each parent. Among our genotypes it varies from 8% for citron to 58% for Brazil Sweet lime (Table 4). Excepted citron, the heterozygosity of other citrus genotypes is higher than 23%. In order to estimate the rate of mappable EST-SSR markers in each putative F1 progeny we have considered the percentage of heterozygous loci, polymorphic and monomorphic between two genotypes in all putative combinations (Table 4). We have not considered *Severinia buxifolia *in this table because it is sexually incompatible with other true citrus varieties and so unsuitable for progeny creation for genetic programs. Heterozygous loci polymorphic between two genotypes could be used as anchored markers suitable for comparative genetic maps (sinteny). Higher values were observed between highly heterozygous species like Morocco sour orange (45%) and Marsh grapefruit (43%) with 29% of loci usable for comparative mapping. At the opposite, whatever the combined genotype, very few loci (less 10%) were available for sinteny in all combinations involving citron that is the less heterozygous citrus specie. In general, combinations including interspecific hybrids such as limes, lemon, grapefruit, orange, and sour orange gave the highest percentage of EST-SSR markers suitable for sinteny (>20%). Kindia combava which is wild citrus specie is heterozygous as interspecific hybrids (43%) and is also characterized by high percentage of suitable markers for sinteny whatever the parental partner excepted with Corsican citron and Pink pummelo. We have estimated also the percentage of monomorphic heterozygous loci (upper part of the table 4). If the allelic segregation could be expected in these loci, the parental origin for inherited allele could not be assigned and then the information related to meiosis in both genotypes is lost. These markers were usually included in segregation data set for genetic map construction from F1 progeny, with the hypothesis of equal recombination rate and normal segregation between male and female genomes. In few combinations the percentage of heterozygous and monomorphic loci is quite high for instance for clementine/sweet orange (20%) or Brazil sweet lime/lemon (21%). Nevertheless, excepted pairs involving clementine and sweet orange combined with Willow leaf mandarin, sour orange and grapefruit, the percentage of heterozygous loci showing a same profile between two genotypes was very low near zero.

**Table 4:** Percentage of heterozygous loci, for each citrus genotype (diagonally bold characters); percentage of monomorphic heterozygous loci between each pair of genotype (italic characters in the upper right size of the table) and percentage of polymorphic heterozygous loci between two genotypes (normal characters in the left down part of the table).

IN*	Varieties	1	2	3	4	5	6	7	8	9	10	11	12	13	14	15
1	Clementine	**36**	*20*	*3*	*18*	*11*	*9*	*0*	*0*	*3*	*6*	*0*	*0*	*0*	*0*	*3*
2	Valencia late sweet orange	3	**40**	*6*	*15*	*11*	*12*	*0*	*0*	*3*	*6*	*0*	*3*	*0*	*0*	*3*
3	Cleopatra mandarin	11	3	**24**	*6*	*3*	*0*	*0*	*0*	*3*	*3*	*0*	*0*	*0*	*0*	*3*
4	Willow leaf mandarin	9	3	12	**43**	*9*	*9*	*0*	*0*	*3*	*3*	*0*	*3*	*0*	*0*	*3*
5	Morocco sour orange	20	23	11	15	**45**	*3*	*0*	*0*	*3*	*6*	*0*	*3*	*0*	*0*	*3*
6	Marsh grapefruit	12	18	12	12	29	**43**	*0*	*3*	*0*	*0*	*0*	*3*	*0*	*0*	*0*
7	Corsican citron	6	3	3	3	3	3	**8**	*3*	*0*	*0*	*3*	*0*	*0*	*0*	*0*
8	Mexican lime	24	21	15	24	24	18	3	**41**	*9*	*6*	*3*	*3*	*3*	*0*	*0*
9	Brazil sweet lime	20	17	11	24	23	21	3	24	**58**	*21*	*6*	*6*	*6*	*0*	*6*
10	Lisbon lemon	21	15	15	24	24	21	3	24	21	**49**	*9*	*3*	*0*	*3*	*6*
11	Sans pepins pummelo	15	15	12	15	18	12	3	15	18	9	**32**	*0*	*0*	*0*	*0*
12	Pink pummelo	6	9	9	9	6	12	3	12	12	9	12	**27**	*0*	*0*	*0*
13	Kindia combava	21	21	15	21	24	18	6	21	24	21	21	9	**43**	*0*	*0*
14	Pomeroy trifoliate orange	11	17	9	12	17	18	3	18	20	9	15	12	21	**29**	*0*
15	Marumi kumquat	11	11	11	9	17	15	3	12	11	9	12	6	15	17	**34**

* Identification number

Estimated percentages of mappable loci in each F1 progeny were presented in Table 5. The mean value of mappable loci calculated on the basis of all results was 57% for this set of genotypes. In details, higher values were observed for different combinations involving Brazil sweet lime with different genotypes, as Marsh grapefruit (80%), Valencia late sweet orange (78%) or Morocco sour orange (77%). It is quite interesting to note that the combination of the two more heterozygous genotypes (Brazil sweet lime and Lisbon lemon) produced a relatively low percentage of mappable marker (65%) due to high level of commune markers (21% of polymorphic plus 21% of monomorphic). The less efficient combination was observed for Corsican citron associated with Cleopatra mandarin (29%) due to the high homozygous level of these genotypes.

**Table 5:** Percentages of mappable loci in each progeny derived from all genotype combinations.

IN*	Varieties	1	2	3	4	5	6	7	8	9	10	11	12	13	14
1	Clementine														
2	Valencia late sweet orange	53													
3	Cleopatra mandarin	46	55												
4	Willow leaf mandarin	52	65	49											
5	Morocco sour orange	50	51	55	64										
6	Marsh grapefruit	58	56	55	65	56									
7	Corsican citron	38	45	29	48	50	48								
8	Mexican lime	53	60	50	60	62	63	43							
9	Brazil sweet lime	71	78	68	74	77	80	63	66						
10	Lisbon lemon	58	68	55	65	64	71	54	60	65					
11	Sans pepins pummelo	53	57	44	60	59	63	34	55	66	63				
12	Pink pummelo	57	55	42	58	63	55	32	53	67	64	47			
13	Kindia combava	58	62	52	65	64	68	45	60	71	71	54	61		
14	Pomeroy trifoliate orange	54	52	44	60	57	54	34	52	67	66	46	44	51	
15	Marumi kumquat	56	60	44	65	59	62	39	63	75	68	54	55	62	46

* Identification number

### EST-SSR markers for genetic diversity analysis

**Figure 4:**
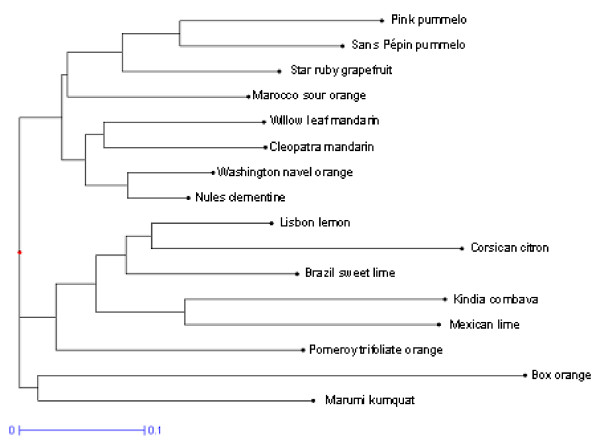
Dendrogram representing the structure of genetic diversity and relationships observed between the 16 citrus genotypes aimed by the polymorphism of the 39 single locus EST-STMS markers.

In order to evaluate the ability of EST-SSR markers to be used for systematic studies a cluster analysis of genetic diversity was done combining polymorphism data of dinucleic and trinucleic EST-SSR (Fig. 4). The sixteen genotypes were clearly differentiated and the relationships between them were organized around two major groups, clearly defined: The first group associated mandarins, orange, sour orange, grapefruit and pummelo. The second one was constituted mainly by the acidic species such as lemon, limes, citron and combava. We can note that trifoliate orange was included in this group even if it represents related genera. The last two genotypes, *Severinia buxifolia *and *Fortunella japonica*, were not included in any genetic clusters.

## Discussion

### Frequency, distribution, and polymorphism

From the 11 391 unigenes obtained from 37 000 EST [19], 1692 microsatellite sequences were identified. 14% of unigenes contain at list one microsatellite as already mentioned for other citrus resources by Chen et al. [23]. This value can be considered as quite high by taking account of the selection pressure that is applied on genes to maintain a lower diversity on the coding region. Nevertheless this frequency is higher than observed for dicotyledonous species ranged between 2.65% and 10.62% [41,42]. The frequency is dependent on the presence or not of redundancy but also related to the parameters used for SSRs screening in the database mining. Varshney et al [43] reported that the frequency was about 5% when the minimum length for the detection of microsatellite was 20 nucleotides. In our study we were less drastic for the detection of SSR. We have fixed this criterion to a minimum of 6 repetitions for dinucleotide repeats (12 base pairs in length) and 5 for the others (15 base pairs in length for trinucleotide). This difference could explain our higher frequency of SSRs in ESTs without apparently any effect on polymorphism (see below). Trinucleotide and dinucleotide repeats were the most common SSRs in clementine ESTs (53.9% and 37.6% respectively). These values reflect the predominance of trinucleotide and dinucleotide repeats in many EST plant species [23,42-46] meanwhile a strong divergence was observed in a hexanucleotide repeat frequency. In many crops they were abundant with a frequency ranged between 13–26%. In clementine ESTs they represent only 2.4% of overall SSRs.

Functional characterization of ESTs performed with GO annotation showed that all the main functional categories were represented. This is in agreement with previous results [18,19]. The EST- SSRs showed similar distributions in the GO Slim categories, and no functional group was overrepresented, indicating that there is no preference in the location of microsatellites with respect to function of the genes.

Relation between SSR polymorphism and phenotypic variation could be investigated in any MIPSs functional categories. Moreover, the EST-SSRs could represent a convenient and cheap way for genes mapping when compared to RFLP technique and sequencing. Unfortunately, the frequency of gene containing a SSR sequence is relatively low (14%). Moreover less than 66% of the analyzed SSR were polymorphic. That means that less than 9% (14% × 66%) of the unigenes should be mapped by internal SSR markers. Seven of the 47 couples of primers amplified DNA fragments from clementine that were larger than expected suggesting the presence of introns. It is possible that the non amplification for the 9 other primers couples was also due to the presence of introns.

From the position analyze of SSRs in ESTs we founded that the majority of SSRs were located in the UTR and mostly (75%) in the first hundred bases of the 5' cDNA extremity. This non equal distribution of SSRs along the cDNA sequence was also reported in other crops such as rice, wheat and barley [17] but with some divergences. In barley, the majority of SSRs are present in the EST 3'-sequences with a high proportion of dimeric and tetrameric SSRs despite tetrameric SSRs are quite absent in Clementine ESTs. As clementine EST clones were single-pass sequenced from their 5' end and their main size were about 800 nucleotides [19], 3'end sequences of these ESTs were certainly under represented. The EST 3' end region was known to be also reached in microsatellites sequences [44,45]. As a consequence of this method of EST production, we believe that we have introduced a bias in the general distribution of the different SSRs along the clementine transcribed sequences. Nevertheless, few works described the abnormally high frequency of microsatellite in 5'UTR regions of plant genes, and a lower abundance in coding region or 3'UTRs [43,45]. Our results seem to confirm this feature. This heterogeneous distribution of SSRs could be explained by the incidence of the SSR variability on the gene transcription and/or proteins structure integrity and function. In UTR, these microsatellites can be more variable without changing gene transcription and translation. The dominance of trimeric SSRs in TR can be explained by the suppression of non-trimeric SSRs in coding regions due to the risk of frame shift mutations that may occur when those microsatellites alternate in size of one unit. In the case of trimeric repeats, it is worth to note that this kind of microsatellite was distributed homogenously along ESTs. It could be hypothesized that trinucleotide SSR variations has less impact than dinucleotide variations in the gene functionality. Indeed, modification of the number of repeats of trinucleotide does not affect the reading frame. Furthermore, dimeric SSRs seem to be more polymorphic than trimeric ones and particularly in UTR with a putative higher allelic diversity combined with higher heterozygosity contributing to a powerful capacity for distinctness. These differences between repeated unit types were attenuated or disappeared when they were located in TR. However, the importance of this result has to be attenuated since we do not have an equal representation for each situation and a too low sampled marker set. Unfortunately, only 4 loci with di-SSRs in TR were detected when compared to 12 for trimeric SSRs and then the differences were not statistically significant.

### EST-SSR markers for citrus diversity

Genetic diversity analysis and systematic is a classical application of SSR markers. For such application, the ability of one marker to differentiate germplasm accessions is an important characteristic. Due to their higher polymorphism, markers localized in UTR are more interesting than markers in TR. Moreover, better rate of accession identification have been obtained with dinucleotide markers (0.61) than with trinucleotide ones (0.29).

The organization of genetic diversity obtained with EST-SSR is in agreement with the knowledge of the genetic relationships between *Citrus *species previously reported by studies using different markers for systematic analyses: morpho-physiological characters [47], biochemistry [48], isozymes [49,50], genomic SSR markers [13,14], CAPS markers [51], or RFLP and RAPD markers [52]. Three major ancestral species: mandarins, pummelos and citrons are at the origin of many cultivated hybrids. As well, the parental relation of limes and lemons with citrons was clearly demonstrated by all these studies. It is in agreement with the strong differentiation we observed between acidic citrus group (lime-lemon-citron) and the pummelo-mandarin (and their hybrids) group. Lemon is thought to be a natural hybrid of a citron and a lime [47,48], or a hybrid of citron and sour orange [51,53]. Our results seem to comfort the participation of sour orange because 15 alleles specific from this genotype were detected in lemon since 10 from citron and only 3 from lime were observed. Nevertheless, we can not certify the parental combination because in our sampling the lime and citron groups were limited to a unique variety. The diversity of these groups were not represented as described previously [13,14] and so few alleles from lemon (4) were still absent in the three putative parents of our study. Several hypotheses have also been proposed to explain the origin of Mexican limes: hybrids of citrons and papedas [48], tri-hybrid cross of citron, pummelo, and *Microcitrus *[47] or hybrid between citron and *C. micrantha *[51]. As for lemon the limited diversity of our analysis does not allow to discuss these hypotheses. Sour orange is a natural hybrid of a mandarin and a pummelo and in our analysis it is associated to the pummelo cluster. The participation of the two basic species, pummelo and mandarin, to the sweet orange formation is attested by the citrus taxonomy literature. However, some troubles still remain concerning the number of crosses between these two basic species. Barkeley et al. [14] suggested that sweet orange was derived from one or more backcrosses to the mandarin and then its genetic was makeup derived from mandarin and a small proportion from pummelo. Nicolosi et al. [51] have proposed a single cross based on equal proportions of alleles from mandarin and pummelo. Our results, with a common cluster of mandarin and sweet orange support the first hypothesis where sweet orange has a higher proportion of alleles from mandarin.

Compared to the phylogeny made with genomic SSR [14] a single difference was observed in our representation. It concerns the genetic diversity between citrus genera. The trifoliate orange (*Poncirus trifoliata*) joins the cluster of citron-limes-lemon while kumquat (*Fortunella japonica*) remains genetically distant to other citrus. In previous work [14] about genetic relationships based on genomic SSRs, the situation was inverted wherein *Fortunella *species were much more closely related to the four other *Citrus *(mandarins, pummelos, citrons and papedas), and the group of *Poncirus *accessions were very distant to all others. This difference could be related by the overrepresentation of kumquat diversity in our study or by a real difference of polymorphism rate between genomic SSRs and EST-SSRs. A similar study on a larger citrus sampling could be suitable to resolve this question. We can not compare the transferability of EST-SSR and genomic SSRs, but a large majority of EST-SSR markers could be used to investigate the genetic of citrus relatives. Indeed, only 10% of those EST-SSR markers gave unsuccessful amplification in Box orange (*Severinia buxifolia*).

### EST-SSR marker for citrus genome mapping

Citrus have a juvenility period with around 5 years of duration limiting the possibility to work on a second generation of hybrids. Consequently a lot of citrus genetic maps are established on F1 progenies at interspecific [25] and intergeneric level [26-31]. In order to evaluate the proportion of mappable EST-SSR markers we have calculated the percentage of heterozygous markers informative for all combinations between 15 sexually compatible citrus genotypes, currently used or susceptible to be used in citrus genetic programs. Table 4 represents a tool for the selection of the sexual cross most suitable for a higher efficiency of mappable markers associated to the better situation for comparison of both parental maps. Higher percentages of markers are available to map secondary species of cultivated citrus than to establish genetic maps of the three basic taxa (citron, mandarin, pummelo). As a result, a very low rate of EST-SSR markers is usable to make comparative genetic mapping between these three basic taxa: it is only 3% for Citron/Pummelo, 3% for Citron/Mandarin (cv Cleopatra) and around 9% for Pummelo (cv Pink)/Mandarin (cv Cleopatra).

It is clear that the best way to map the higher number of markers in a single progeny is to work on segregation of interspecific or intergeneric crosses. *Citrus *× *Poncirus *progenies have been highly investigated [11,54-59]. A recent work on EST genetic maps for *Citrus sinensis *and *Poncirus trifoliata *was published [59]. For these maps the authors have studied the segregation of 300 pairs of primers generating EST-SSR markers on the intergeneric progeny sweet orange × trifoliate orange. Among them 141 markers (47%) were mapped and distributed as following: 122 markers (40.7%) on sweet orange map, 59 (19.7%) on trifoliate orange one and 40 (13.3%) were commune to both. These values were very similar to those proposed in our work (table 4 and 5) where for the same parental cross we have estimated at 52% of of mappable EST-SSR markers and 40%, 29% and 17% respectively for orange, trifoliate orange maps and commune markers. This mapping work was done with a majority of non abundant SSRs in ESTs such as compound, tetra-, penta- and hexa-nucleotide repeats. Di and tri-nucleotide SSRs represent only 26.7% of the total studied SSR markers.

On the base of the genetic differentiation observed in our cluster analysis, it appears that in this frame, interesting progenies should be obtained from F1 hybrids between citron and pummelo, citron and mandarin, as well between poncirus or kumquat with citron or mandarin or pummelo. Such intrageneric progenies should probably have more interest for further QTLs analysis of quality traits.

## Conclusion

We have observed a differential repartition of dinucleic and trinucleic SSRs in the clementine ESTs with a high concentration in UTR and more precisely in the 5'initial region (but without a default of representation of 3'UTR regions du to the strategy of EST sequencing). The degree of SSR polymorphism is strongly modified by the utility of coding regions. These two elements suggest that the natural selection should limit the number and the polymorphism of SSRs in coding translated sequences. EST-SSRs are useful for enhancing individual species map, but can be used as anchor probes for creating links between maps in comparative studies. With the appropriate progeny arise from crosses between interspecific or intergeneric hybrids as parents, we can expect to use up to 80% of the EST-SSR markers representing 9% of the global set of genes from all the identified function groups. We suggest to focus on the dinucleotide SSRs localised in UTR (more heterozygous and polymorphic) to increase the efficiency of mapping loci and then to reduce the cost of molecular marker screening between the parents of a progeny. In addition to mapping ESTs via microsatellite loci for locating putative functions, the EST-SSR markers developed in this study are powerful for the study of genetic diversity of citrus.

## Authors' contributions

FLL carried out molecular genetic studies, data analysis and drafted the manuscript. GC and TA participated to generate results on genotyping. JT and XA generated the EST data base and did the annotation. PW and MT, were in charge of the clementine EST analysis in the framework Genoscope project. PO and RM coordinated the Genoscope project and participated to the drafting of the manuscript.
